# Gene array analysis of adrenal glands in broiler chickens following ACTH treatment

**DOI:** 10.1186/1471-2164-10-430

**Published:** 2009-09-14

**Authors:** Clara Bureau, Christelle Hennequet-Antier, Michel Couty, Daniel Guémené

**Affiliations:** 1 UR83-Unité de Recherches Avicoles, Institut National de la Recherche Agronomique, Centre de Tours-Nouzilly, 37380 Nouzilly, France

## Abstract

**Background:**

Difference in adaptability responses to stress has been observed amongst bird species, strains, and individuals. Components of the HPA axis, one of the internal systems involved in homeostasis re-establishment following stress, could play a role in this variability of responses. The aim of the present study was 1) to identify genes involved in the regulation of adrenal activity following ACTH stimulation and 2) to examine adrenal genes differentially expressed in individuals with high and low plasma corticosterone response following ACTH treatment.

**Results:**

Analysis with 21 K poultry oligo microarrays indicated that ACTH treatment affected the expression of 134 genes. Several transcripts assigned to genes involved in the adrenal ACTH signaling pathway and steroidogenic enzymes were identified as differentially expressed by ACTH treatment. Real-time PCR on 18 selected genes confirmed changes in transcript levels of 11 genes, including MC2R, CREM, Cry, Bmal1, Sqle, Prax1, and StAR. Only 4 genes revealed to be differentially expressed between higher and lower adrenal responders to ACTH treatment.

**Conclusion:**

The results from the present study reveal putative candidate genes; their role in regulation of adrenal functions and adaptability to stress should be further investigated.

## Background

Farm animals are often confronted with various challenging situations that may have a negative impact on their performance [1]. Under stressful conditions, several systems, mainly the hypothalamic-pituitary-adrenal (HPA) axis, the autonomic nervous system (ANS) [2,3], and the immune system [4] are called into action to re-establish homeostastis. This is done by setting up a cascade of regulatory mechanisms [5,6], resulting in a mobilization of energy and a shift in metabolism with detrimental effects on growth performance and efficiency of feed utilization. The large variability across species, breeds and individuals in their responses to stress challenges and variability appears to have a large genetic component [7-12]. In fact, the response of adrenal glands to ACTH seems to be variable in numerous species, including rat [27], pig [11,13-16] and quail [10,17] and difference in adrenal responsiveness may play a role in the variability of the response to stress.

For example, in a pioneering series of studies with pigs, Hennessy et al. [18] showed that the adrenal response to ACTH is an individual characteristic, which is heritable and reproducible across successive testing [18,14,19]. In broilers, studies indicated that plasma corticosterone concentration after stress is an inheritable trait [20]. Numerous behavioral traits, such as feeding behavior, feather pecking or stress susceptibility, are also heritable [21]. Koolhaas et al. [22] also evoked the possible implication of genetics in the stress coping strategy of animals.

The HPA axis is a system mainly involved in the long-term stress response of the animal and allows the organism to deal with stress without exhausting internal resources. The hypothalamus synthesizes neuropeptides, mainly vasopressin (AVP) or vasotocin (AVT), its equivalent in birds, and corticotrophin releasing hormone (CRH), which triggers the release of ACTH from the pituitary gland [23]. ACTH acts on adrenal glands stimulating the release in the blood circulation of cortisol (or corticosterone in birds). There is evidence that the hypothalamus, the pituitary gland or the adrenal glands, could play a pivotal role on the variability of the response to stress. Previous studies have suggested that the functional differences in HPA axis have a central origin [24-27], mainly due to differences in hypothalamic CRH neuron function [28]. Other studies have reported changes occurring at the pituitary [29] or adrenal cortex levels [13].

The present study aimed to investigate the molecular genetic regulation of the sensitivity of the adrenal glands to ACTH in broiler chickens. This was achieved by the study of gene expression in the adrenal glands under basal conditions and in response to ACTH treatment using a microarray approach followed by validation by quantitative RT-PCR. Corticosterone levels upon ACTH treatment were also determined and two groups of chickens with high or low response to ACTH were constituted and their gene expressions compared.

## Methods

### Animals and housing

Fifty-four Ross PM3 female chickens were used in this study. The experiment was conducted at the Unité expérimentale Pôle d'Expérimentation Avicole de Tours (UEPEAT - INRA, Tours, France) according to the legislation on research involving animal subjects set by the European Community Council Directive of November 24, 1986 (86/609/EEC). One-week-old chickens were housed in individual cages, and kept on 16 h of light and 8 h of dark with feed and water provided *ad libitum*. The animals were reared under these standard conditions for a period of three weeks.

### Treatment and sampling

At 28 days of age, the animals were either non-treated (Control, n = 27) or injected (Treated, n = 27) in the pectoralis major muscle with the mammalian 1-24 ACTH (Immediate Synacthène, Novartis, France) at 50 μg/kg of body weight and returned to their cages before being sacrificed. In order to have the most accurate basal conditions, the non-treated animals were not injected with a vehicle. The birds were sacrificed immediately after capture (Control), or 1 h following treatment. Blood samples were collected directly from the wing vein in pre-heparinized syringes from all animals just before being sacrificed. Samples were centrifuged at 2000 g for 15 min at 4°C and the plasma was separated and stored at -20°C until measurement of corticosterone using a specific radio immunoassay [30]. The adrenal glands were collected, deep frozen in liquid nitrogen before being stored at -80°C until RNA isolation.

### Microarray analysis

RNA was extracted from the entire adrenal glands using the Nucleo Spin RNA-L extraction kit (Marcherey-Nagel, France) and according to manufacturer's instruction. The amount and the purity of RNAs were analyzed using the NanoDrop^® ^ND-1000 UV-Vis Spectrophotometer (Palaiseau, France) and the integrity of RNA was assessed by using Agilent 2100 bioanalyzer (Montlucon, France). Gene expression profiling was performed with chicken oligo microarrays (Ultragaps, Corning). Corning slide microarrays were obtained from INRA-GADIE (Jouy-en-Josas, France) Resource Center . A set of 21 120 oligonucleotides (70 mers) were spotted onto each slide. Oligo probes were designed by Roslin Institute/ARK Genomics and synthesized by Operon (Ebersberg, Germany). More details about this oligonucleotide set as well as further gene annotation and informatics can be found at the Operon's website .

On the basis of the corticosterone measurement for treated group, we reconstituted two sub-groups of eight (8) animals each, one regrouping animals with the highest levels of corticosterone and the other with the lowest levels of corticosterone. From the control group, eight (8) animals were randomly selected to perform microarray analysis. For this, two experiments were performed. In Experiment 1, we compared the control (n = 8) with the treated group that showed a high CORT (n = 8). In the other experiment (Experiment 2), we compared animals with high CORT (n = 8) to those with a low CORT (n = 8). RNAs from the individuals from the group with the high response to treatment were used for both experiments. For each sample, an amount of 4 ug of total RNA (24 samples) was amplified using the Amino Allyl Message Amp a RNA kit (Ambion) according to the protocol provided by the kit. Following amplification, two (2) batches of 10 μg of the obtained aaRNA from each sample were labeled, one with the Alexa Fluor 555 and the other one using Alexa Fluor 647 (Fisher Scientific, France). A total of 32 microarray slides (16 for each study) were hybridized using a dye-swap method (each couple of aaRNA being compared twice by inversing dyes each time).

Thirty (30) picomoles of each labeled probe were used for hybridization. The arrays were hybridized using Pronto Corning kit following the manufacturer's protocol (Life Science).

### Microarray Signal Processing and statistical analysis

Arrays were scanned using GenePix 4000B scanner and GenePix Pro software . Spots with low oligonucleotide signals (lower than the background level) were excluded from the analysis. Statistical analyses were performed using the R software (version 2.7.2, [31]) and Anapuce R package. A normalization consisting of a global lowest regression was applied on the overall log ratios to remove the bias due to fluorescence incorporation. A block effect was also considered by subtracting the median value of the block. A differential analysis based on a paired t-test assuming a specific variance by gene was performed. A gene was declared differentially expressed if its adjusted p-value by Bonferroni method was lesser than 5% [32].

### Functional annotation

Transcripts significantly affected by ACTH treatment were annotated for their function according to Gene Ontology database [33]. Functional enrichment analysis of up-regulated and down-regulated genes was performed using FATIGO  and overrepresentation of gene ontology categories was assessed by means of a Fisher's exact test.

### Analysis of RNA changes by relative quantitative real-time PCR

To validate changes in gene expression from the microarray analysis results, real-time PCR was carried out on 18 selected genes, chosen on the basis of their high expression rate (fold change), for their specific function related to cholesterol or steroid synthesis and cholesterol transfer, and some also chosen since their function remain unknown to this day. An amount of 5 μg of total RNA from each sample (the same one used to perform microarray analysis) was reverse transcribed in a total volume of 20 μl using 200 UI of Superscript II (Invitrogen) reverse transcriptase, 250 ng random primers, 0.5 mM deoxy-NTP, and 40 U of RNAse Out (Invitrogen). The resultant cDNA was diluted 1:100 with nuclease-free water. Five microliters of diluted cDNA was used in subsequent PCR reactions. Primers were designed based on nucleotide sequences in Genebank using the Primer Express software (PE Applied Biosystems) see Table 1. The PCR reaction consisted of 1.6 × Power SYBER Green PCR Master Mix (PE Applied Biosystems), 0.5 μM forward and reverse primers and 5 μL of the diluted cDNA to a total volume of 20 μl. Reactions were carried out on an ABI PRISM 7500 Sequence detection system (PE Applied Biosystems) for 40 cycles (95°C for 15 s, 60°C for 1 min). The fold change in expression of each gene was calculated using the CT method with the levels of transketolase RNA as an internal control; determined by quantitative RT-PCR. The levels of transketolase did not change depending on treatment in our study (data not shown) and transketolase gene has previously been used to normalize data from quantitative RT-PCR in adrenal cells [34]. Quantitative real-time PCR analysis was performed in triplicate. ANOVA was conducted on relative expression to assess the effects of treatment and the intensity of response.

**Table 1:** Gene specific primers used for quantitative RT-PCR

**Gene symbol**	**Forward Primer**	**Reverse Primer**	**Accession Number**
ARL10	GCTGCCTGTTCCATCCTCAT	CCTTACCCCAAGTGCTCCC	XM_414552
d-cry	TACACTGCCGTGGGTGGTACT	GCCCCCACATCTAAGCCTG	NM_205501
Predicted PRAX-1	GGAAACTCGCTGCCAAACA	CCTACTGGCGTTGCGTTGA	XM_001236228
TSKU	GCTCAGCTGGGATCGTGTG	ATCCTTCCCGTGATAGGCAA	NM_001005346
ICER	GTAATGGCAGCCTCCCCAG	GGCAGCTTCCCTGTTTTTCA	NM_204387
Predicted HSD17B7	TACCACCAACCTCTTTGGGC	TGCTGGAAGAGGTCCAGATGA	XM_001232165
MYPN	ATCCTCCACCTGCACAATCG	AGACAGCGTTAATGGGACCG	XM_421565
Predicted KCTD1	CCCCTGCATCTCCATTGAAC	GGTATACATGTGCCCACCCAC	XM_001231830
Predicted NARFL	CCTGACTGGATGCCCTTGAA	GCAGCCCT GGAGATAATGTTG	XM_414836
Predicted NUDT5	GAGAGTGCTGCATTGCGAGAG	TGCTGTTTGACACACCTGGG	XM_001235499
Predicted DNAJA5	TGGGAGAAACGAGCTATGGAA	TCCCTCTTGCGAATAAAGGC	XM_425006
Predicted CCNG1	GATCGAGTCTGCCCATGACAA	TGTGGAAGCCGAAGAACTGA	XM_414493
STAR	AGTGATGGCCCTTATCTCGGT	GGTGGCTGCTACAAACACTGC	NM_204686
SC4MOL	CACATCCTCTGGAAACGCTCA	GCGACATATTACCCATGCCC	NM_001006438
NCB5R	CCTACCACCACTATTCCCCCA	GCGACACTATGGCACACCC	XM_416445
PISD	CGTGCAGTTTGCCAAGTCCTA	CCTGAACAGCCACCATCACC	XM_415253
ATF4	CATGGGTTCTCCAGCGACA	GAGAAGGCATCCTCCTGGCT	NM_204880
FKBP5	CCTCCAATGCCACCCTCTTT	TCCTTTCCTCTTGATCCTCCG	NM_001005431
Transketolase	CGAGTGATTGCTTTGGATGGA	TCACGAGTGGCACAACCAACT	XM_414333

## Results

### Corticosterone levels

**Figure 1:**
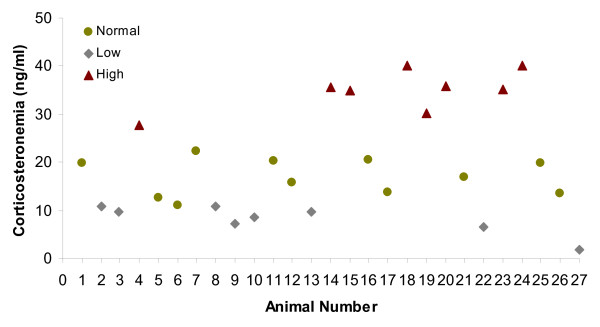
**Plasma corticosterone pattern in ACTH treated chickens**.

Animals treated with 1-24 ACTH showed higher plasma corticosterone levels (p < 0.0001) compared to non-treated ones. In the treated group, the corticosteronemia values varied between animals (Figure 1) and averaged 18 (± 10) ng/ml. The corticosteronemia for control animals averaged 2 (± 1) ng/ml.

### Gene transcript regulation by ACTH treatment

After normalization, 7657 of 21120 oligonucleotides present on the chicken oligo microarray (36%) were found to be expressed in adrenal glands in our experimental conditions. Among them 134 and 4 transcripts appeared to be differentially expressed in Experiments 1 and 2, respectively. A single transcript (predicted PRAX-1) was found to be up-regulated in both experiments, with the group with a higher response to treatment showed a higher expression for that transcript compared to the control group (Experiment 1) or to the lower response to 1-24 ACTH injection (Experiment 2). Several transcripts assigned to genes involved in the adrenal ACTH signaling pathway and steroidogenic enzymes were identified as differentially expressed, including HSD17B7, IDI1, HMCS1, SC4MOL, PRAX-1, StAR, SQLE, BMAL, NCB5R, MC2R and ICER. Other transcripts with yet unknown function, and others involved in transcription activity, cell division, signal transduction, electron transfer, and other assigned functions were identified. Full details in gene name, function, accession number, fold change and p-value for all transcripts differentially expressed are provided as an additional file (Additional file 1).

Functional enrichment analysis of gene up-regulated by ACTH treatment indicated significant enrichment of expression of gene involved in biogenic amine metabolic process (GO:0006576), steroid metabolic process (GO:0008202), and amino acid derivative biosynthetic process (GO:0042398). Analysis of genes down-regulated by ACTH treatment indicated signficiant enrichment of gene involved in response to protein stimulus (GO:0051789), and response to temperature stimulus (GO:0009266).

### Quantitative analysis by real time PCR

Following microarray analysis, a total of 14 genes and all 4 transcripts found differentially expressed from Experiments 1 and 2, respectively, were further analyzed by RT-PCR. Changes in transcript levels were confirmed in 11 genes (nine (9) from Experiment 1 and two (2) from Experiment 2). Table 2 summarizes the result obtained by RT-PCR

**Table 2:** Differential gene expression by microarray and RT-PCR analysis

	**Microarray analysis**		**RT-PCR**		
**Sequence**	**Fold H/C**		**Fold (H/C)**	**Fold (L/C)**	**Fold (H/L)**

Genes up-regulated by ACTH treatment					

ARL10	7	H = L > C	9	4	NS
ASAL	5.6	H = L > C	31	7	NS
PRAX-1	3.8	H = L > C	5	5	NS
C-TSK	3.4	H > L > C	57	14	4
ICER	3.4	H > C, C = L, L = H	12	NS	NS
HSD17B7	3.2	H > C, H > L, C = L	14	NS	4
STAR	2.3	H = L = C	NS	NS	NS
SC4MOL	1.5	H = L = C	NS	NS	NS
PISD	2.6	H = L > C	3	3	NS
ATF4	1.5	H = L = C	NS	NS	NS
FKBP	1.4	H = L = C	NS	NS	NS

Genes down-regulated by ACTH treatment					

MYPN	3.2	H < L, H < C, L = C	15	7	NS
KCTD1	2.3	H < L, H < C, L = C	3	NS	3
NARFL	2	H = L = C	NS	NS	NS
NCB5R	1.4	H = L, H < C, L = C	2	NS	NS

Genes differentially expressed in high and low ACTH response groups					

NUDT5	0.65	H = L = C	NS	NS	NS
AGT2	0.53	H = C = L, L > C	NS	2	NS
CCNG1	1.23	H = L = C	NS	NS	NS
PRAX-1	1.32	H = L, H > C, L > C	5	5	NS

NS: No statistical significant difference is shown between compared groups

H: High responder group

L: Low responder group

C: Control group

## Discussion

The objective of this present study was to seek insights into the genetic basis of the variability of response to stress in chickens. Corticosteronemia following ACTH injection varied between animals. Individual variations in adrenal responses have already been described in previous study [35]. This variation can, in part, be related to time course responses to ACTH injection. According to Noirault et al. 1999 [35], a maximum response is generally achieved 15 min post-injection and lasts for a period of 5 to 20 min, then drops gradually to reach the basal level. In our study, the blood was taken one hour post treatment, therefore animals with the highest level of CORT can be considered either having a high adrenal response or a prolonged response, and the opposite (low adrenal response or short delay response) for those with low level of CORT.

Following microarray analysis, changes in the expression of many genes encoding proteins involved in the response of adrenal glands to ACTH stimulation were observed. To our knowledge, this is the first study investigating gene expression in adrenal tissues following ACTH treatment in birds. A total of 134 transcripts in adrenal glands revealed to be affected by acute ACTH treatment. As expected, the expression level of several genes encoding steroidogenic enzymes and biogenic amines metabolism were significantly affected by ACTH treatment. However, genes commonly described to respond to ACTH treatment with mammals [36-38], such as genes belonging to the P450 family (P450 11A1, P450 11B1, P450 11B2, P450 c17 and P450 c21) or the 3β hydroxysteroid dehydrogenase (3β HSD) family, were not found differentially expressed in response to ACTH treatment in the present study with birds. Our results suggest that another member of cytochrome P450 family (CYP51), and another member of the HSDs family (17 β HSD type 7) appeared to be activated in response to ACTH treatment in the chicken. CYP51 or the lanosterol 14alpha-demethylase, is known to be the only cytochrome P450 (P450) that is widely conserved from prokaryotes to eukaryotes, and is believed to represent the ancestral P450 [39]. In eukaryotes, CYP51 catalyzes the 14α-demethylation of 14α-methylsterol, which is an essential process in cholesterol biosynthesis [40]. Up to eight (8) isoforms of 17 β HSDs have been identified, but their roles in steroid metabolism remain unclear [41]. The regulation of all HSDs is controlled by trophic hormones and the signaling mechanisms involve cAMP-dependent protein kinases and protein kinase C, but the exact mechanisms by which this leads to altered gene transcription have not been clearly elucidated.

Data on key regulatory genes involved in the steroidogenic pathway are lacking in chickens. Most of data available are from studies carried out with mammals. It is hypothesized that gene expression steroidogenic enzymes following ACTH treatment is only significantly modulated during long-term stimulation. There seems to be only limited effect on adrenal P450s and steroidogenic enzymes gene expression in response to acute ACTH treatment [11,42,43]. Based on previous work from our laboratory [35], the dose of ACTH used in the present study should maximally activate corticosterone production as early as 15 min following ACTH treatment and this high response can last up to one hour post-treatment. Therefore, it is not clear whether the lack of modulation of expression of genes belonging to P450 family (P450 11A1, P450 11B1, P450 11B2, P450 c17 and P450 c21) or the 3β hydroxysteroid dehydrogenase (3β HSD) in the present study is due to the nature of stimulation (acute with long duration) or related to the fact that different genes (such as CYP51 and 17 β HSD type 7) may be responsive in chickens compared to mammals.

In the present study, IPKG was activated by ACTH treatment. IPKG is known to be a potent competitive inhibitor of cAMP-dependent protein kinase activity [44]. It is generally recognized that in mammals, cAMP dependent transcription is the most important level of regulation of steroidogenic genes [45]. As such, IPKG could be an important regulator of steroid hormones production in animals under stressful conditions.

Up-regulation of StAR (steroidogenic acute regulatory protein) in treated animals compared to controls was observed in the present study. StAR is involved in cholesterol transport into mitochondria [46]. The up-regulation of StAR in the present study supports previous studies which indicated that a rapid increase in StAR mRNA levels, and steroid hormone production, is observable in the steroidogenic cells of the adrenal cortex in response to acute ACTH/cAMP stimulation [47,48]. An up-regulation of ICER (inducible cAMP early repressor) in response to ACTH treatment was also observed in the present study. ICER is involved in the regulation of the cAMP-dependent transcription of StAR [49]. However, both StAR and ICER were not found to be differentially expressed between the higher and lower responders to ACTH treatment groups in Experiment 2. However, results from microarray analysis revealed an up-regulation of PRAX-1 (peripheral-type benzodiazepine receptor associated protein 1) in animals with higher adrenal response compared to the lower responders to ACTH treatment. This finding failed to be confirmed by RT-PCR analysis probably because the fold-change was not high enough to be detected. However an up-regulation of PRAX-1 in the treated groups (either with high or low response) compared to the control was observed following RT-PCR analysis. PRAX-1 is involved in steroid hormone synthesis by assuring the transport of cholesterol into the mitochondria [50]. This suggests that enhanced cholesterol transport into mitochondria might contribute to the higher corticosteroid biosynthesis found in animals with a higher response to ACTH treatment. This finding is consistent with what was observed in pigs in a previous study [11].

Several other genes were found in the present work to be affected by ACTH treatment. Among those, Sqle (squalene epoxidase) which is involved in endogenous cellular cholesterol synthesis [51] or Cry (delta 2 crystallin) and Bmal (Aryl hydrocarbon receptor nuclear translocator-like protein) which are known as peripheral clock genes. Both Cry and Bma1 regulate a large number of genes involved in general cellular processes as well as in pathways related to major organ-specific function, such as, corticosteroid biosynthesis. These genes may be important regulators of adrenal sensitivity to ACTH [52,53]. In the case of Sqle and Cry, both were up-regulated but Bmal was down-regulated by ACTH. A large number of other genes encoding for protein kinases, protein phosphatases and trancriptional factors were also found to be differentially expressed in the present study. Their involvements in adrenal steroidogenesis have not been characterized and deserve some attention, in particular C-TSK whose expression level was positively influenced by ACTH treatment even in animals from the group with the low adrenal response to ACTH.

## Conclusion

Analysis of gene expression in adrenal tissue in response to ACTH stimulation in chickens highlighted several responsive genes. Many of these genes have already been described to play an important role in steroidogenesis regulation. A number of genes that had not been directly implicated in ACTH-steroid pathway were also highlighted. Their contributions to adrenal function deserve further investigations.

The major mechanism explaining differences between animals with high or low response to ACTH treatments may be in the transport of cholesterol into the mitochondria for steroidogenesis with PRAX-1 is putatively a good candidate to target the sensitivity of animals to stress.

## Abbreviations

ACTH: adrenocorticotropic hormone; ARL10: ADP-ribosylation factor-like 10; Bmal: Aryl hydrocarbon receptor nuclear translocator-like protein; d-cry: delta 2 crystallin; HMCS: 1Hydroxymethylglutaryl-CoA synthase; HSD17B7: hydroxysteroid (17-beta) dehydrogenase 7; ICER: inducible cAMP early repressor; IPKG: cAMP-dependent protein kinase inhibitor gamma; MC2R: melanocortin 2 receptor; NCB5R: NADH-cytochrome b5 reductase; PRAX-1: peripheral-type benzodiazepine receptor-associated protein 1; SC4MOL: sterol-C4-methyl oxidase-like; StAR: steroidogenic acute regulator; SQLE: squalene epoxidase.

## Authors' contributions

CB participated in the design of the study, carried out the molecular genetic studies, and drafted the manuscript. CH performed the statistical analysis. MC carried out CORT assay and participated in tissues sampling. DG conceived the study, participated in its design and coordination, and helped to draft the manuscript. All authors read and approved the final manuscript.

## Supplementary Material

Additional file 1 **Complete list of genes differentially expressed between animals**. The data provided represent a complete list of genes differentially expressed between animals a) with high (H) response to treatment and control (C) and b) with high (H) versus low (L) response to ACTH treatment. Click here for file
