# Supplementary material for: Gene array analysis of adrenal glands in broiler chickens following ACTH treatment
Source: BMC Genomics. 2009 Sep 14;10:430. doi: 10.1186/1471-2164-10-430 (PMC2751787; doi:10.1186/1471-2164-10-430)
Supplement: Additional file 1 — Complete list of genes differentially expressed between animals. The data provided represent a complete list of genes differentially expressed between animals a) with high (H) response to treatment and control (C) and b) with high (H) versus low (L) response to ACTH treatment. [file 1471-2164-10-430-S1.doc]

Additional file 1.

Complete list of genes differentially expressed between animals :

a) with high (H) response to treatment and control (C)

| **Gene**  **symbol** | **Gene name** | **Gene Ontology Terms** | **Accession Nº (Genbank)** | **Fold change (H/C)** | **p-value** |
| --- | --- | --- | --- | --- | --- |
| **Gene upregulated by ACTH treatment** | | | | | |
| NR0B1 | Nuclear receptor subfamily 0, group B, member 1 | Adrenal gland development | NM_204593| | 1.9 | 2.79 E-03 |
| NR5A1 | Nuclear receptor subfamily 5, group A, member 1 | Adrenal gland development | NM_205077 | 1.9 | 4.40E-03 |
| IFRD1 | Interferon-related developmental regulator 1 | Adult somatic muscle development | NM_001001468| | 1.6 | 3.14E-02 |
| ASAL (dCry) | Argininosuccinate lyase | Amine metabolic process | NM_205501 | 5.6 | 6.05E-04 |
| GS | Glutamine synthetase | Amino acid metabolic process | NM_205493 | 1.4 | 1.31E-02 |
| NCOA4 | Nuclear receptor coactivator 4 | Androgen receptor signaling pathway | NM_001006495 | 2 | 1.18E-05 |
| ARL10 | ADP-ribosylation factor-like protein 10 | Biological process unknown | XM_414552 | 7.1 | 3.60E-05 |
| SC4MOL | sterol-C4-methyl oxidase-like | C4 methylsterol oxydase activity | NM_001006438 | 1.5 | 5.37E-03 |
| AMYP | Pancreatic alpha-amylase | Carbohydrate metabolic process | NM_001001473 | 1.7 | 6.24E-03 |
| MK12 | Mitogen-activated protein kinase 12 | Cell cycle arrest | XM_001233061 | 2.5 | 4.78E-04 |
| BCAR1 | Breast cancer anti-estrogen resistance 1 | Cell motion | XM_414057 | 2.1 | 5.54E-03 |
| Trx | Thioredoxin | Cell redox homeostasis | NM_205453 | 1.5 | 1.26E-03 |
| SQLE | Squalene epoxidase | Cellular aromatic component metabolic process | NM_001030953| | 1.5 | 3.60E-02 |
| PISD | Phosphatidylserine decarboxylase proenzyme | Cellular component | XM_415253 | 2.6 | 9.31E-05 |
| RIOK2 | RIO kinase 2 RIOK2 | Cellular component | NM_001006581| | 1.5 | 2.07E-02 |
| CLTA | Clathrin, light chain (Lca) | Cellular component organization | NM_001039313 | 1.3 | 2.23E-02 |
| StAR | Steroidogenic acute regulatory protein | Cellular lipid metabolic process | NM_204686| | 2.3 | 2.94E-02 |
| ATF4 | Activating transcription factor 4 | Cellular metabolic process | NM_204880| | 1.5 | 3.12E-04 |
| CMBL | Carboxymethylenebutenolidase homolog (Pseudomonas), transcript variant | Cellular metabolic process | XM_001231950 | 3.7 | 1.55E-04 |
| PAICS | Phosphoribosylaminoimidazole carboxylase, phosphoribosylaminoimidazole succinocarboxamide synthetase | Cellular metabolic process | NM_205524 | 1.5 | 3.17E-02 |
| CYP51 | cytochrome P450, family 51, subfamily A, polypeptide 1 | Cholesterol biosynthetic process | NM_001048077| | 1.7 | 4.67E-03 |
| HSD17B7 | 17-beta-hydroxysteroid dehydrogenase 7 | Cholesterol biosynthetic process | XM_001232165 | 3.2 | 6.04E-05 |
| IDI1 | Isopentenyl-diphosphate Delta-isomerase 1 | Cholesterol biosynthetic process | XM_418561 | 3.6 | 1.91E-03 |
| RERE | Arginine-glutamic acid dipeptide (RE) repeats | Chromatin remodeling | XM_417594 | 1.7 | 2.70E-04 |
| HMCS1 | Hydroxymethylglutaryl-CoA synthase | Cytoplasm | NM_205411 | 1.9 | 5.08E-03 |
| AMD1 | S-adenosylmethionine decarboxylase 1 | Embryonic development | NM_001012569| | 2.3 | 4.56E-05 |
| MK09 | Mitogen-activated protein kinase 9 | Embryonic development | NM_205095| | 1.4 | 2.75E-03 |
| MBNL | Muscle blind-like protein | Embryonic limb morphogenesis | NM_001012573 | 1.4 | 1.31E-02 |
| CL004 | Chromosome 12 open reading frame 4 | Enerrgy reserve metabolic process | XM_001231609 | 2.4 | 2.26E-03 |
| SAR1A | Similar to SAR1a protein | Establishment of localization | XM_421589| | 1.4 | 3.42E-02 |
| Gal 10 | Gallinacin 8 prepropeptide (Beta-defensin 10) | Galactose catabolic process | NM_001001609 | 1.4 | 2.54E-02 |
| UAP1 | UDP-N-acteylglucosamine pyrophosphorylase 1 | Glucosamine metabolic process | XR_027008 | 2.4 | 9.68E-03 |
| ICER | Inducible cAMP early repressor | Glycolipid metabolic process | NP_989718 | 3.2 | 3.34E-02 |
| ALAS1 | Amino-levulinate, delta-, synthase 1 | Heme biosynthesis process | NM_001018012| | 2.1 | 4.89E-03 |
| PSIP1 | PC4 and SFRS1 interacting protein 1 | Initiation of viral infection | NM_001031610 | 2.7 | 3.68E-04 |
| PTPN2 | Protein tyrosine phosphatase, non-receptor type 2 | Insulin receptor signaling pathway | XM_419126 | 1.7 | 1.98E-03 |
| STK6 | Serine/threonine-protein kinase 6 | Mitotic cell cycle | XM_425725 | 1.9 | 3.32E-04 |
| HNRPDL | Heterogeneous nuclear ribonucleoprotein D-like | mRNA metabolic process | NM_001031142| | 1.2 | 1.10E-02 |
| GADD45 | Growth arrest and DNA-damage-inducible protein | Negative regulation of protein kinase activity | | 3.3 | 4.95E-05 |
| EGR-1 | Early growth response protein 1 | Negative regulation of transcription | NM_204136 | 1.5 | 3.65E-02 |
| TOB1 | transducer of ERBB2, 1 | Negative regulation of transforming growth factor beta receptor signaling pathway | NM_001001467| | 1.5 | 1.74E-02 |
| SLITRK4 | Similar to neuronal transmembrane protein Slitrk4 | Neuron projection morphogenesis | XM_420266 | 1.9 | 9.80E-03 |
| NUBP2 | nucleotide binding protein 2 | Nucleoside-triphosphatase activity | NM_001007833 | 1.5 | 8.73E-04 |
| AZIN1 | Antizyme inhibitor 1 | Polyamine metabolic process | NM_001008729 | 1.4 | 1.01E-02 |
| SKP2 | S-phase kinase-associated protein 2 | Positive regulation of estrogen receptor signaling pathway | NM_001007982 | 1.6 | 6.12E-04 |
| CITED4 | Cbp/p300-interacting transactivator; with Glu/Asp-rich carboxy-terminal domain; 4 | Positive regulation of transcription | NM_204718 | 2 | 3.79E-04 |
| PTN2 | Tyrosine-protein phosphatase non-receptor type 2 | Protein amino acid dephosphorylation | |XM_419126 | 1.8 | 1.15E-02 |
| ALG9 | Asparagine-linked glycosylation protein 9 homolog | Protein amino acid glycosylation | XM_001235479| | 1.4 | 2.57E-02 |
| FKBP5 | FK506-binding protein 5 | Protein binding | NM_001005431| | 1.4 | 1.87E-02 |
| PDLI4 | PDZ and LIM domain protein 4 | Protein binding |  | 1.5 | 3.81E-03 |
| AHSA1 | Activator of 90 kDa heat shock protein ATPase homolog 1 | Protein folding | XM_001233709 | 1.4 | 9.39E-03 |
| Hsp60 | Heat shock protein; mitochondrial precursor | Protein folding | NM_001012916 | 1.4 | 2.18E-03 |
| HSPA8 | Heat shock cognate 71 kDa protein | Protein folding |  | 1.6 | 5.75E-05 |
| HSPA8 | Heat shock cognate 71 kDa protein | Protein folding |  | 1.6 | 5.75E-05 |
| IPKG | cAMP-dependent protein kinase inhibitor gamma | Protein kinase regulation activity | XM_001232747 | 1.5 | 2.77E-02 |
| eRF1 | Eukaryotic peptide chain release factor subunit 1 | Protein metabolic process | XM_414511| | 1.7 | 9.39E-03 |
| ETF1 | Eukaryotic peptide chain release factor subunit 1 | Protein metabolic process | XM_414511 | 1.6 | 2.70E-03 |
| FBXW11 | F-box and WD-40 domain protein 11 | Protein metabolic process | NM_001039262 | 1.9 | 1.44E-04 |
| TFPI2 | Tissue factor pathway inhibitor 2 precursor | Proteinaceous extracellular matrix | XM_418662 | 1.8 | 1.06E-02 |
| CHST12 | Carbohydrate sulfotransferase 12 | Proteoglycan biosynthetic process | XM_414775 | 1.7 | 4.42E-03 |
| ADCY5 | Adenylate cyclase type 5 | Regulation of adenylate cyclase activity involved in G protein activity | | 1.7 | 9.12E-05 |
| SAT1 | Diamine acetyltransferase 1 | Regulation of cell proliferation | NM_204186 | 1.2 | 4.54E-02 |
| PEDF | Pigment epithelium-derived factor precursor | Regulation of neurogenesis | XM_001234864 | 1.4 | 1.58E-03 |
| MCL-1 | Myeloid cell leukemia | Response to cytokin stimulus | XM_001233734 | 2.2 | 3.74E-02 |
| SMAD 6 | Mothers against decapentaplegic homolog 6 | Response to stimulus | NM_204248 | 1.6 | 1.06E-02 |
| CIRBP | Cold inducible RNA binding protein | Response to stress | NM_001031347 | 1.4 | 1.14E-02 |
| GNAI3 | Guanine nucleotide-binding protein G(k) subunit alpha (G(i) alpha-3 | Signal transduction | NM_204249 | 1.4 | 1.79E-03 |
| MC2R | Melanocortin 2 receptor | Signal transduction | NM_001031515 | 1.6 | 5.34E-03 |
| TSPAN6 | Tetraspanin 6 | Signal transduction | XM_420247 | 1.4 | 9.69E-03 |
| C-TSK | Tsukushi precursor | Skeletal muscle fiber development | NM_001005346 | 3.4 | 2.88E-03 |
| XBP1 | X box-binding protein 1 | Tissue morphogenesis | NM_001006192 | 1.8 | 1.49E-03 |
| DDX26B | DEAD/H (Asp-Glu-Ala-Asp/His) box polypeptide 26B | Unknown | XM_420227| | 2.6 | 1.33E-05 |
| FBXO34 | F-box protein 34 | Unknown | NM_001031213 | 1.4 | 3.49E-02 |
| MSPD1 | Motile sperm domain-containing protein 1 | Unknown | XM_420226 | 2.1 | 9.63E-04 |
| NT5DC2 | 5'-nucleotidase domain containing 2 | Unknown | XM_414247 | 1.3 | 1.69E-02 |
| PRAX-1 | Peripheral-type benzodiazepine receptor-associated protein 1 | Unknown | XM_001236228 | 3.8 | 8.76E-03 |
| Similar to calcium binding protein P22 | Similar to calcium binding protein P22 | Unknown | NM_001007930 | 1.3 | 6.38E-03 |
| TOR2A | Torsin-2A precursor | Unknown | XM_415532 | 0.4 | 1.43E-04 |
| Unknown | Unknown | Unknown | XM_420263 | 3.1 | 2.03E-02 |
| Unknown | Unknown | Unknown | XM_001231609 | 3.1 | 7.21E-03 |
| Unknown | Unknown | Unknown | XM_421913 | 2.3 | 1.61E-02 |
| Unknown | Unknown | Unknown | NM_001030704 | 2.2 | 4.14E-02 |
| Unknown | Unknown | Unknown | NM_001031011 | 2 | 2.04E-02 |
| Unknown | Unknown | Unknown | NM_204737| | 1.9 | 6.84E-03 |
| Unknown | Unknown | Unknown | XM_001233451 | 1.7 | 5.77E-03 |
| Unknown | Unknown | Unknown | XM_001234072 | 1.7 | 4.82E-02 |
| Unknown | Unknown | Unknown | XM_419295 | 1.6 | 6.29E-03 |
| Unknown | Unknown | Unknown | NM_001079726 | 1.6 | 8.91E-03 |
| Unknown | Unknown | Unknown | XM_001232691 | 1.6 | 1.16E-02 |
| Unknown | Unknown | Unknown | NM_001039298 | 1.6 | 3.54E-02 |
| Unknown |  | Unknown | XM_001232607 | 1.5 | 1.01E-04 |
| Unknown | Unknown | Unknown | XM_001236877 | 1.5 | 1.22E-03 |
| Unknown | Unknown | Unknown | XM_001231686| | 1.4 | 3.40E-02 |
| Unknown | similar to ribonuclease P 14kDa subunit | Unknown | XM_414402 | 1.7 | 3.61E-02 |
| WIPI3 | WD repeat domain phosphoinositide-interacting protein 3 | Unknown | NM_001007844 | 1.4 | 2.38E-03 |
|  | |  |  |  |  |
| **Genes down-regulated by ACTH treatment** | | | | | |
| BPGM | 2,3-bisphosphoglycerate mutase BPGM | Carbohydrate metabolic process | NM_001030768 | -1.5 | 3.02E-03 |
| ASB9 | Ankyrin repeat and SOCS box-containing 9 | Cellular component | NM_001006262 | -1.4 | 2.86E-02 |
| TRAP1 | TNF receptor-associated protein 1 | Cellular developmental process | NM_001006175 | -1.3 | 3.79E-02 |
| HspB1 | Heat-shock protein beta-1 | Contractile fiber | NM_205290 | -1.3 | 1.23E-02 |
| similar to NSE1 | Similar to NSE1 | DNA repair | XM_419958 | -1.6 | 1.41E-02 |
| Pod-1 | Transcription factor 21 (Podocyte-expressed 1 | Embryonic development | XM_419734 | -1.4 | 3.71E-02 |
| SURB7 | SRB7 suppressor of RNA polymerase B | Embryonic development | XM_416442 | -1.4 | 2.52E-02 |
| similar to MutS homolog 4 | Similar to MutS homolog 4 | Female gamete generation | XM_422549 | -1.5 | 7.48E-03 |
| PHKB | Phosphorylase kinase, beta | Generation of precursor metabolites | NM_001007831 | -1.2 | 1.96E-02 |
| ITPK1 | Inositol-tetrakisphosphate 1-kinase | Inositol phosphate-mediated signaling | XM_430052 | -1.2 | 3.60E-02 |
| CTSA | Cathepsin A | Intracellular protein transport | NM_001031491 | -1.1 | 2.15E-02 |
| VAMP | Vesicle-associated membrane protein | Intracellular protein transport | NM_001006296 | -1.2 | 2.32E-02 |
| NCB5R | NADH-cytochrome b5 reductase | Mitochondrial envelope lumen | XM_416445 | -1.4 | 6.27E-03 |
| NARFL | Nuclear prelamin A recognition factor-like | Negative regulation of transcription factor activity | XM_414836 | -2.1 | 1.21E-02 |
| KCTD1 | Potassium channel tetramerisation domain containing 1 | Nervous system development | XM_001231830| | -2.3 | 9.68E-04 |
| RPIA | Ribose 5-phosphate isomerase A | No biological data base | NM_001031170 | -1.2 | 9.91E-03 |
| COMMD5 | COMM domain containing 5 | No biological data base | NM_001008465 | -1.3 | 3.86E-02 |
| similar to RIKEN cDNA | Similar to RIKEN cDNA | No biological data base | XM_001232398 | -1.8 | 4.20E-03 |
| ZWILCH | Zwilch, kinetochore associated, homolog | No biological data base | XM_413922| | -1.3 | 1.50E-02 |
| SLU7 | Pre-mRNA-splicing factor SLU7 | Nuclear mRNA splicing | NM_001006146 | -1.3 | 3.46E-02 |
| NENF | Neuron-derived neurotrophic factor | Positive regulation of MAPKKK cascade | XM_419430 | -1.2 | 1.35E-02 |
| LAMA2 | Laminin alpha-2 chain precursor | Positive regulation of synaptic transmission, cholinergic | XM_419746 | -1.2 | 3.63E-03 |
| GATA5 | Transcription factor GATA-5 (GATA-binding factor 5) | Regulation of transcription | NM_205421 | -1.8 | 2.74E-04 |
| BMAL2 | Aryl hydrocarbon receptor nuclear translocator-like protein 2 | Regulation of transcription, circadien rhythm | NM_204133 | -1.4 | 2.92E-02 |
| NDUFA1 | NADH dehydrogenase [ubiquinone] 1 alpha subcomplex subunit 1 | Response to chemical stimuli | XM_001234261 | -1.2 | 2.56E-03 |
| GPR22 | Probable G-protein coupled receptor 22 | Signal transduction | XM_001231968 | -1.6 | 5.83E-04 |
| MPPED1 | Metallophosphoesterase domain containing 1 | Unknown | XM_416454 | -1.3 | 1.61E-02 |
| MYPN | Myopalladin | Unknown | XM_421565 | -3.2 | 4.37E-04 |
| Unknown | Unknown | Unknown | XM_416707 | -1.2 | 4.92E-02 |
| Unknown | Unknown | Unknown | XM_417419 | -1.3 | 2.76E-02 |
| Unknown | Unknown | Unknown | NM_001007841 | -1.2 | 1.40E-02 |
| Unknown | Unknown | Unknown | NM_001031014 | -1.4 | 2.52E-02 |
| Unknown | Unknown | Unknown | XM_001234194 | -1.4 | 1.50E-02 |
| Unknown | Unknown | Unknown | XM_417124 | -1.4 | 2.23E-03 |
| Unknown | Unknown | Unknown | XM_001232954 | -1.8 | 1.00E-03 |
| WDR22 | WD repeat protein 22 | Unknown | XM_426432 | -1.4 | 4.17E-02 |

**b) with high (H) versus low (L) response to ACTH treatment**

| **Gene Symbol** | **Gene name** | **Function** | **Accession Nº (Genbank)** | **Fold change (H/L)** | **p-value** |
| --- | --- | --- | --- | --- | --- |
| **Genes up-regulated** | |  |  |  |  |
| PRAX-1 | Peripheral-type benzodiazepine receptor-associated protein 1 | Regulation of cholesterol transfert | XM_001236228 | 1.3 | 5.21E-03 |
| CCNG1 | cyclin G1 | Cell division | XM_414493 | 1.2 | 7.24E-04 |
| **Genes down-regulated** | |  |  |  |  |
| NUDT5 | Nucleoside diphosphate-linked moiety X motif 5 | ADP reductase activity | XM_001235499 | -1.5 | 8.01E-03 |
| DNAJA5 | DnaJ homology subfamily A member 5 | May act as a co-chaperone for HSP70 | XM_425006 | -1.9 | 4.86E-02 |
